# Myofibrillogenesis regulator 1 (MR-1) is a novel biomarker and potential therapeutic target for human ovarian cancer

**DOI:** 10.1186/1471-2407-11-270

**Published:** 2011-06-25

**Authors:** Renquan Lu, Min Sun, Jingjing Feng, Xiang Gao, Lin Guo

**Affiliations:** 1 Department of Clinical Laboratory, Shanghai Cancer Center, Fudan University, Shanghai, China; 2 Department of Oncology, Shanghai Medical College, Fudan University, Shanghai, China; 3 Department of Gynecological Oncology, Shanghai Cancer Center, Fudan University, Shanghai, China

**Keywords:** Myofibrillogenesis regulator 1, ovarian cancer, proliferation, invasion, apoptosis

## Abstract

**Background:**

Myofibrillogenesis regulator 1 (MR-1) is overexpressed in human cancer cells and plays an essential role in cancer cell growth. However, the significance of MR-1 in human ovarian cancer has not yet been explored. The aim of this study was to examine whether MR-1 is a predictor of ovarian cancer and its value as a therapeutic target in ovarian cancer patients.

**Methods:**

Reverse-transcription polymerase chain reaction (PCR) and quantitative real-time PCR were used to detect MR-1 mRNA levels in tissue samples from 26 ovarian cancer patients and 25 controls with benign ovarian disease. Anti-MR-1 polyclonal antibodies were prepared, tested by ELISA and western blotting, and then used for immunohistochemical analysis of the tissue samples. Adhesion and invasion of 292T cells was also examined after transfection of a pMX-MR-1 plasmid. Knockdown of MR-1 expression was achieved after stable transfection of SKOV3 cells with a short hairpin DNA pGPU6/GFP/Neo plasmid against the MR-1 gene. In addition, SKOV3 cells were treated with paclitaxel and carboplatin, and a potential role for MR-1 as a therapeutic target was evaluated.

**Results:**

MR-1 was overexpressed in ovarian cancer tissues and SKOV3 cells. 293T cells overexpressed MR-1, and cellular spread and invasion were enhanced after transfection of the pMX-MR-1 plasmid, suggesting that MR-1 is critical for ovarian cancer cell growth. Knockdown of MR-1 expression inhibited cell adhesion and invasion, and treatment with anti-cancer drugs decreased its expression in cancer cells. Taken together, these results provide the first evidence of the cellular and molecular mechanisms by which MR-1 might serve as a novel biological marker and potential therapeutic target for ovarian cancer.

**Conclusions:**

MR-1 may be a biomarker for diagnosis of ovarian cancer. It may also be useful for monitoring of the effects of anti-cancer therapies. Further studies are needed to clarify whether MR-1 is an early diagnostic marker for ovarian cancer and a possible therapeutic target.

## Background

Ovarian cancer is the leading cause of cancer-related death in women worldwide. The American Cancer Society revealed that 21,550 women in the US were diagnosed with ovarian cancer and 14,600 women died of the disease in 2009 [1]. In China, the number of patients with ovarian cancer has increased in recent years, and the 5-year survival rate is less than 30%. Metastasis is the major cause of disease progression and therapeutic failure [2].

Myosin light chain-2 (MLC2) plays an important role in cell migration from solid tumors such as ovarian cancer, and its dephosphorylation can induce apoptosis [3,4]. A recent report indicated that MLC2 may regulate cell proliferation and migration by interacting with myofibrillogenesis regulator 1 (MR-1) [5]. Overexpression of MR-1 is associated with cancer cell proliferation and migration in human hepatoma HepG2 cells [6]. MR-1, mapped to 2q35 and first cloned from a human skeletal muscle cDNA library using PCR and rapid amplification of cDNA ends (Genbank™ accession no. AF417001), is a protein of 142 amino acids [7-10]. MR-1 may promote cancer cell proliferation by binding to specific proteins, such as eukaryon initiation factor 3, which is highly associated with the regulation of tumor cell growth and invasion [11]. Also, overexpression of MR-1 can activate the nuclear factor κB signaling pathway, which is linked to a wide variety of diseases including cancer, inflammation and autoimmune disease [12].

Taking all the evidence into account, we hypothesized that MR-1 may play a role in the development and progression of ovarian cancer, probably by promoting cell proliferation and invasion. The present study examined MR-1 expression in ovarian cancer tissues and a cancer cell line. Overexpression or knockdown of MR-1 in cancer cells was used to assess its role in cell proliferation, adhesion, and invasion. Finally, the response of MR-1 to treatment with anti-cancer drugs was assessed to identify whether it functions as a novel biological marker and therapeutic target for ovarian cancer.

## Methods

### Human Tissue Samples and Cell Lines

All human samples were collected in compliance with the guidelines of the Ethics Committee at the Fudan University Cancer Hospital. Fresh-frozen surgical ovarian tissue samples were collected from 26 patients with ovarian cancer (aged 20-58 years) and 20 control patients with benign ovarian disease (aged 23-55 years) who were admitted to Fudan University Cancer Hospital (Shanghai, China) between July 2008 and December 2009. All cases were confirmed by pathology. The samples were defatted, immediately cut into pieces of appropriate size on ice, and stored at -80°C for later use. The ovarian carcinoma cell line, SKOV3, and the non-ovarian cancer cell line, 293T, were generous gifts from Dr. Meiqin Zhang, Laboratory of Gynecologic Oncology, Fudan University Shanghai Cancer Center, Shanghai.

### Reverse-Transcription Polymerase Chain Reaction (RT-PCR) and Quantitative Real-Time PCR

Total RNA was extracted from the cells using the TRIzol reagent (Invitrogen, NY, USA) according to the manufacturer's protocol, and the RNA concentration was determined using a Nanodrop spectrophotometer (Thermo Scientific, Wilmington, DE, USA). RT-PCR was carried out using a SuperScript™ one-step RT-PCR kit (Invitrogen) according to the manufacturer's instructions. cDNA was synthesized from 2 μg of total RNA at 42°C for 45 min, followed by inactivation of the reverse transcriptase at 85°C for 5 min. The cDNA was then subjected to PCR to amplify the MR-1 and GAPDH (control) genes in 50 μL reaction mixture containing the following primers: MR-1 forward 5'-CCCAGAAAGAGGG GCAAGA-3' (P1), MR-1 reverse 5'-TGAGGATGAAGAG GAGGATACCA-3' (P2), GAPDH forward 5'-GGGAGCCAAAAGGGTCATCATCTC-3' (P1) GAPDH reverse 5'-CCATGCCAGTGAGCTTC CCGTTC-3' (P2). The cycling conditions were: initial denaturation at 95°C for 3 min, followed by 36 cycles of denaturation at 94°C for 30 s, annealing at 55°C for 30 s, and extension at 72°C for 45 s. PCR products were resolved and analyzed on a 2% TAE agarose gel containing ethidium bromide. Quantitative real-time PCR for MR-1 (GAPDH as internal control) was performed in an MJ PCR system (Reno, NV, USA) using a 20 μL reaction volume containing 10 μL of SYBR^® ^Premix Ex Taq™ reagent (Takara, Dalian, China), 0.5 pmol of each primer, 1 μg of cDNA, and the same sets of primers and cDNAs. The conditions were: an initial denaturation step at 94°C for 10 min, followed by 40 cycles of 15 s at 95°C (denaturation), 30 s at 55°C (annealing), and 30 s at 72°C (extension). Delta Ct (Ct_MR-1_-Ct_GAPDH_) was calculated for each set of samples and compared.

### Vector Construction and Production of MR-1 Recombinant Protein

Human MR-1 cDNA was synthesized by reverse transcription from the mRNA (sequence originally published by Li et al [7]). A 429-bp fragment was amplified from the synthesized cDNA by PCR using *Taq *PCRx^® ^DNA polymerase (Invitrogen) to introduce BamHI and XhoI restriction sites using the primers 5'-CGGGATCCATGGCGGCGGTGGTAGCTGC-3' (P3, BamHI site underlined) and 5'-CCGCTCGAGTCAGGTCTGCACCCCAGAC-3' (P4, XhoI site underlined). The cDNA fragment was then cloned into the PET21a (+) expression vector (Invitrogen) to yield PET21a-MR1, which was then transformed into *Escherichia coli *BL21 (DE3) cells for expression of the MR-1 recombinant protein [13]. The MR-1 recombinant protein contained a T7-tag at the N-terminus and 6 × His-tag at the C-terminus and was purified using Ni-resin affinity chromatography. The purified protein was confirmed by western blotting with a mouse anti-His PcAb (Invitrogen).

### Antibody Production

New Zealand white rabbits were immunized four times with the MR-1 recombinant protein. Sera were harvested 3 days after the final immunization if the rabbits showed a good anti-MR-1-His titer. The anti-MR-1 antibodies R1 and R2 were then affinity-purified using a protein A column (Pierce Biotechnology Inc., Rockford, IL, USA). The antibody titers were measured using an enzyme-linked immunosorbent assay (ELISA), and immunoreactivity was confirmed by western blotting with MR-1 recombinant protein and human ovarian cancer cells (SKOV3).

### Immunocytochemistry

Immunohistochemical staining was performed on 6-μm sections from formalin-fixed, paraffin-embedded human tissues. The slides were deparaffinized and rehydrated in graded ethanol solutions. Antigen retrieval was performed by heating the samples in a microwave oven for 20 min in citrate buffer (pH 6.0). The slides were then incubated with the affinity-purified MR-1 antibody (1:200) followed by incubation with a horseradish peroxidase-labeled polymer conjugated to a goat anti-rabbit immunoglobulin antibody (1:4000; R&D Systems, Minneapolis, MN, USA). Color was developed using diaminobenzidine as a chromogen and the slides were counterstained with hematoxylin. An anti-His antibody and polyclonal non-immune rabbit IgG were used as negative controls to check the specificity of the MR-1 affinity-purified antibody. Immunocytochemical staining of SKOV3 cells was performed on cover slips by fixing the cells for 20 minutes in acetone, blocking with 2% bovine serum albumin, and then incubating with affinity-purified anti-MR1 antibody followed by DAB staining and hematoxylin counterstaining. Dark brown cytoplasmic staining was defined as positive and no (or faint) staining was defined as negative. The proportion of tumor cells positive for MR-1 varied considerably: diffuse positive (> 50%), focal or heterogeneous (10%-50%), and trace (< 10%).

### Establishment of Stable Cell Line Overexpressing MR-1

The full-length coding sequence for the MR-1 cDNA was subcloned into the pMX-puro(+) vector (Invitrogen) to yield pMX-MR-1, which was then transfected into 293T cells using Fusion 6^® ^(Roche, Mannheim, Germany) according to the manufacturer's instructions. A pMX-mock vector (pMX-puro(+) containing a random non-MR-1 DNA sequence) was used as the control. Puromycin (0.5 μg/ml; Invitrogen) was added to the cells 48 h after transfection to select stably transfected clones. Single clones were picked from both pMX-MR-1 and pMX-mock transfected samples, and RNA and protein were extracted to check the expression levels of MR-1 by real-time PCR and western blotting. Positive clones were designated pMX-MR-1-293T, and control clones as pMX-mock-293T. 293T cells were cultured in Dulbecco modified Eagle medium (DMEM) supplemented with high glucose, 10% fetal bovine serum (FBS, Gibco BRL, Grand Island, NY, USA), and 1% penicillin/streptomycin at 37°C in 5% CO_2_.

### Cell Proliferation and Invasion Assays

For the cell proliferation assay, pMX-MR-1-293T and pMX-mock-293T cells were grown at a density of 1000 cells/ml in 24-well plates with regular medium changes. The cell number was counted every 24 hours for 6 days as follows: cells were detached by brief exposure to 0.025% trypsin containing 2 mM EDTA in PBS, washed in culture medium without FBS, and then resuspended in the same medium for manual cell counting. For the invasion assays, Matrigel was thawed at 4°C overnight, diluted in cold serum-free culture medium, plated onto 24-well plates preloaded with Transwell™ culture inserts (12 mm diameter, 8 μm pore size; Costar, Cambridge, MA, USA) and incubated for 5 hours at 37°C for gelling. The cells were then plated onto the Transwell™ inserts (50,000 cells/well) and cultured at 37°C in 5% CO_2_. After 16 hours, the cells on the upper side of the well were removed and fixed prior to staining with hematoxylin and eosin. Cells migrating to the underside were counted under a microscope. All experiments were repeated at least three times.

### MR-1 Knockdown Using RNAi

SKOV3 cells were cultured in Gibco^® ^RPMI 1640 (Invitrogen) supplemented with 10% heat-inactivated FBS and 1% penicillin/streptomycin at 37°C in a humidified atmosphere containing 5% CO_2_. When the cells reached 70-80% confluence, they were transfected with pGPU6-MR-1 short hairpin DNA (shDNA) targeting MR-1. The sequences of the shDNAs were as follows: shDNA 232: 5'-CACCGCTAACAAGGCTTCTCATAACTTCAAGAGAGT TATGAGAAGCCTTGTTAGCTTTTTG-3'; shDNA 442: 5'-CACCGACCGTGTGA AGCAGATGAAGTTCAAGAGACTTCATCTGCTTCACACGGTCTTTTTTG-3'; shDNA 584: 5'-CACCGACCCTCCTAGGCTATTGACTTTCAAGAGAAGTCAATA GCCTAGGAGGGTCTTTTTTG-3'; mock sequence: 5'-CACCGTTCTCCGAACGT GTCACGTCAAGAGATTACGTGAGACGTTCGGAGAATTTTTTG-3'. The pGPU6-shDNA plasmids were constructed by cloning the respective shDNAs into the pGPU6/GFP/Neo vector (Invitrogen). The cells were exposed to 1500 μg/ml G418 (Invitrogen) for 2 days after transfection to select for stably transfected clones. Cells were then plated at a lower density in 96-well plates containing RPMI 1640 and G418 (750 μg/ml) until single colonies were formed. Colonies from the SKOV3-MR-1-shDNA and SKOV3-mock-shDNA-transfecetd cells were then picked and expanded. Real-time PCR and western blotting were used to test the expression levels of MR-1. GAPDH was used as the internal control.

### Effects of Anti-Tumor Drugs on MR-1 Expression

SKOV3 cells (50,000 cells/well) were cultured in six-well plates in RPMI 1640 under the conditions described above. After 24 hours, 10 μL of serially diluted paclitaxel (equivalent to 0, 12.5, 25, 50, 100 nmol/L; Bristol-Myers Squibb Inc., USA) or carboplatin (equivalent to 0, 40, 80, 160, 320 mg/L) (Qilu Pharmacy Inc., China) were added to the medium and the cells were cultured for an additional 48 hours. One set of cells was treated with TUNEL reagent (Roche) and counterstained with DAPI, and the number of apoptotic cells (stained by Annexin V-FITC/PI) was determined by flow cytometry (Becton Dickinson, USA). The other set of cells was harvested for RNA and protein extraction, and the expression levels of MR-1 were determined by real-time RT-PCR and western blotting.

### Statistical analysis

Statistical analysis was performed using Analysis of Variance (ANOVA) and the post-hoc Student-Newman-Keuls test, with SPSS software version 13.0 (SPSS Inc., Chicago, IL, USA). The results were expressed as the mean ± SD. A *P *value < 0.05 was considered significant.

## Results

### MR-1 Is Differentially Expressed in Human Malignant and Benign Ovarian Tumors

**Figure 1:**
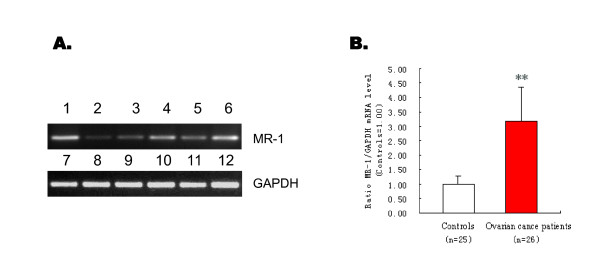
**MR-1 mRNA levels detected by RT-PCR and real-time quantitative PCR**. (A) RT-PCR products from cells and tissue samples from patients with ovarian cancer were visualized on 2% agarose gels. *Upper panel*: MR-1 RT-PCR products from SKOV3 cells (Lane 1), two samples from patients with benign ovarian disease (Lanes 2, 3), and three samples from patients with ovarian cancer (Lanes 4, 5, 6). *Lower panel*: GAPDH RT-PCR controls for the above samples in the same order (Lanes 7-12). (B) Real-time quantitative PCR of ovarian cancer patients (*n *= 26) showed increased MR-1 mRNA levels compared with controls. **P < 0.05.

Tissue MR-1 mRNA levels were analyzed by RT-PCR and quantitative real-time PCR to investigate the expression of MR-1 in malignant and benign ovarian tumors. RT-PCR showed that the mRNA expression level was higher in ovarian cancer patients than in controls (Figure 1A). Quantitative real-time PCR also showed that MR-1 expression was significantly higher in tumor tissues from ovarian cancer patients (*n *= 26) than in control patients (*n *= 25) (*P *< 0.05; Figure 1B). Western blotting showed a higher level of MR-1 protein expression in ovarian cancer tissues.

### Production of MR-1 PcAbs

**Figure 2:**
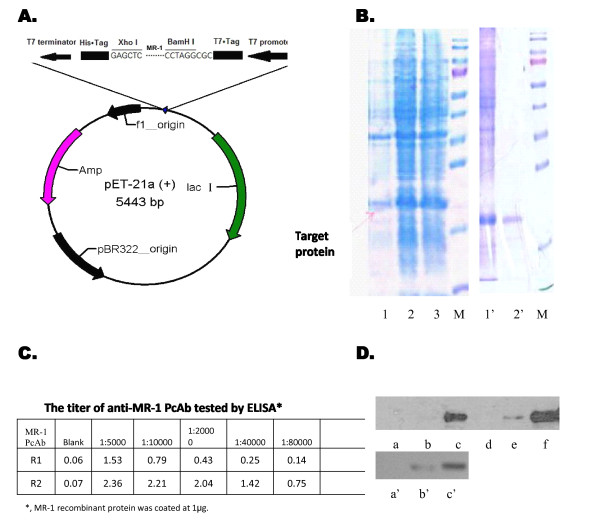
**Preparation of MR-1 recombinant protein and anti-MR-1 polyclonal antibodies (PcAbs)**. (A) Construction of the of MR-1 expression plasmid. (B) The induced and purified MR-1 recombinant protein was visualized by 15% sodium dodecylsulfonate-polyacrylate gel electrophoresis (SDS-PAGE). Lanes 1-3: expressed products of MR-1 recombinant protein at 0 h, 2 h and 4 h; Lane 1': unpurified MR-1 protein; Lane 2': purified MR-1 protein; M: protein molecular weight ladder (170, 130, 100, 70, 55, 40, 35, 25, 15, 10 kDa). (C) The titer of anti-MR-1 PcAbs was tested by ELISA (*R1*: > 1:40,000; *R2*: > 1:80,000). (D) Western blotting of the MR-1 protein. *Upper panel*: MR-1 recombinant protein at 0 μg (a, d), 0.1 μg (b, e), 1 μg (c, f) was detected by anti-MR-1 PcAbs R1 and R2 at a dilution of 1:2000. *Lower panel*: Total protein from SKOV3 cell lysates at 0 μg (a'), 1 μg (b') and 10 μg (c') was detected by anti-MR-1 PcAb R2 at a dilution of 1:2000.

Immunization of rabbits with MR-1 recombinant proteins (Figure 2A) successfully yielded anti-MR-1 antibodies, which were then affinity-purified (Figure 2B). The PcAbs, named R1 and R2, were further assessed by ELISA and western blotting. The results showed that both antibodies, particularly R2, reacted with the MR-1 protein, and had a titer of around 1:5 × 10^5 ^(Figure 2C and 2D).

### Overexpression of MR-1 in Ovarian Cancer

**Figure 3:**
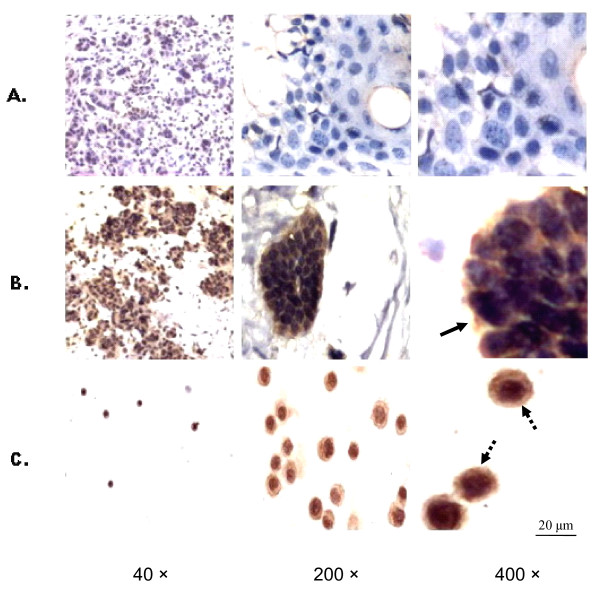
**Expression of MR-1 protein in human ovarian tissues and ovarian cancer cell lines (Left to right: magnification = × 40, ×200, and × 400)**. (A) Ovarian epithelial cells from cysts were negative for MR-1. (B) Serous papillary ovarian carcinoma cells stained positive for MR-1 in the cytoplasm and nuclei (bold arrow). (C) SKOV-3 cells showed strong cytoplasmic and nuclear staining for MR-1 (dashed arrow).

Immunohistochemical staining of 26 ovarian cancer tissue samples and 20 ovarian cyst samples showed that the MR-1 protein was located in both the cytoplasm and nuclei of ovarian carcinoma cells, whereas the epithelial cells of the cysts were negative (Figure 3A and 3B). Diffuse staining for MR-1 was observed in ovarian cancer cells, particularly serous papillary ovarian cancer cells, compared with the benign control group. Approximately 73% (19/26) of ovarian carcinoma samples showed diffuse positive staining, 19% (5/26) showed focal or heterogeneous staining, and 8% (2/26) showed trace staining. The control anti-His antibody and the purified non-immune rabbit IgG showed negative staining (data not shown). High MR-1 expression and a similar staining pattern were detected in SKOV3 cells (Figure 3C).

### MR-1 Overexpression Promotes Cell Adhesion and Invasion

**Figure 4:**
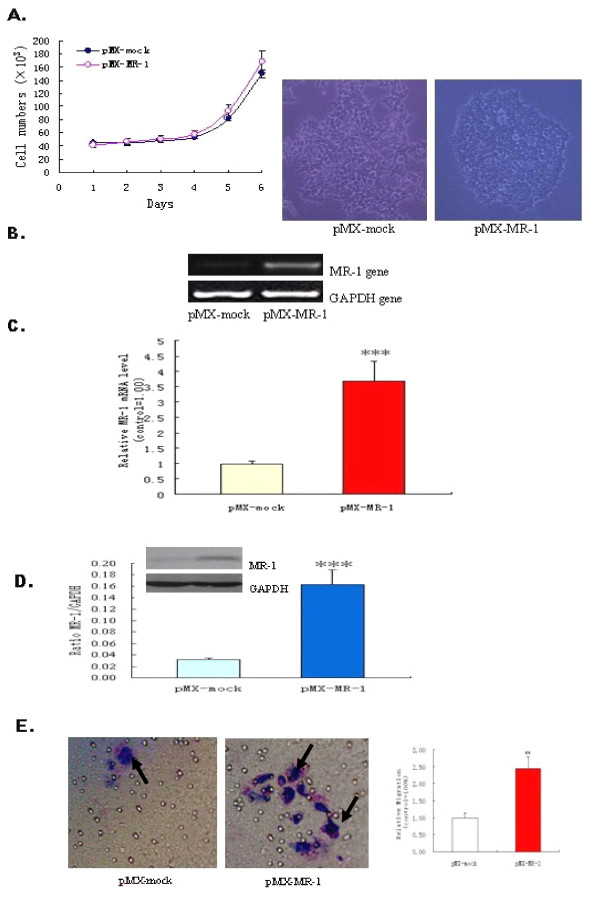
**MR-1 expression in 293T-pMXs-MR-1 and 293T-pMX cells**. (A) Cell proliferation was assessed by cell counting, and cell growth was observed under an optical microscope. (B, C) 293T-pMX-MR-1 cells showed increased MR-1 levels as detected by RT-PCR and real-time quantitative PCR. (D) Western blotting showed increased levels of MR-1 protein expression in 293T-pMX-MR-1 cells. (E) Representative images of migrated cells (left: × 400, arrow). Invasiveness of 293T was enhanced by transfection of the MR-1 gene (right). All experiments were repeated at least three times. **P < 0.05; ***P < 0.01 compared with pMX-mock control.

This is the first report describing the generation of pMX-mock- and pMX-MR-1-stably-transfected 293T cells. Tests were conducted to ensure that overexpression of MR-1 did not affect proliferation of the 293T cells; however, the density of 293T-pMX-MR-1 cells was higher (Figure 4A). RT-PCR and real-time PCR revealed that pMX-MR-1-stably-transfected 293T cells had much higher levels of MR-1 mRNA expression than control pMX-mock-stably-transfected 293T cells (Figure 4B and 4C). This was mirrored by protein expression levels as assessed by western blotting (Figure 4D). pMX-MR-1-stably-transfected 293T cells appeared to aggregate to a greater extent than pMX-mock-stably-transfected 293T cells, indicating enhanced cell adhesion, which is a key step in the cell invasion process. As cells adhere and spread, they generate traction and thereby migrate through the substrate. The results of the Transwell assays showed that transfection with pMX-MR-1 significantly promoted the invasion of 293T cells (Figure 4E).

### Effects of MR-1 Knockdown on Cell Proliferation, Adhesion and Spreading

**Figure 5:**
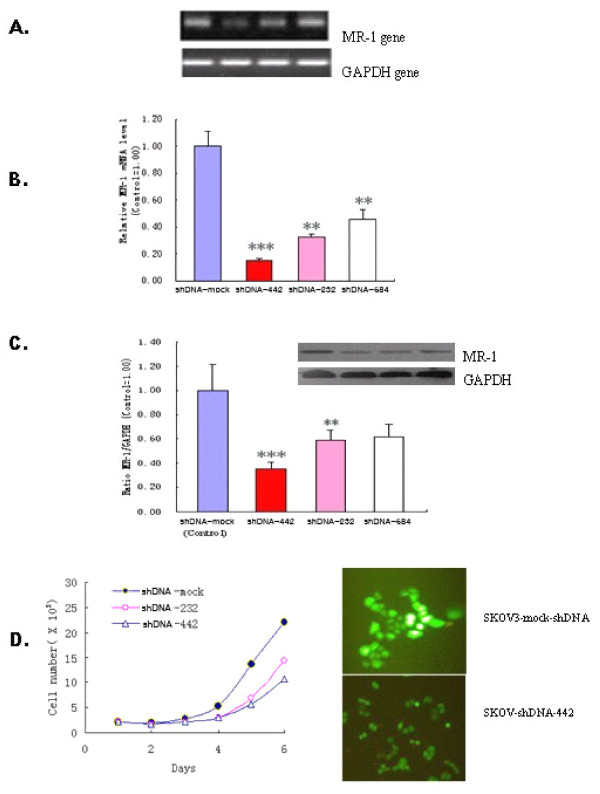
**Inhibition of cell proliferation by down-regulating MR-1**. (A) RT-PCR showed that expression of the MR-1 gene decreased in SKOV3 cells transduced with MR-1 ShDNA-442, ShDNA-232, ShDNA-584 (left to right: ShDNA-mock, ShDNA-442, -232, and -584). (B) Expression of the MR-1 gene was markedly reduced in SKOV3 cells transfected with ShDNA-442, -232, and -584, (particularly with ShDNA-442) as analyzed by two-way ANOVA. (C) MR-1 protein levels were also decreased in SKOV3 cells transfected with ShDNA-442, and -232 (but not ShDNA-584). ***P < 0.01, **P < 0.05 compared with shDNA-mock controls. (D) SKOV3 cells transfected with ShDNA-442, and -232 showed inhibited proliferation and delayed spreading (left). Reduced expression of MR-1 protein fused to GFP in SKOV3 transfected with ShDNA-442 (right).

SKOV3 cells constitutively overexpress MR-1. To further investigate the function of MR-1, we constructed MR-1-shDNA recombinant plasmids and stably transfected them into SKOV3 cells to knockdown the expression of MR-1. RT-PCR, real-time PCR and western blotting showed that MR-1 expression was dramatically down-regulated at both the mRNA and protein levels in SKOV3-MR-1-shDNA-442 and -232 cells (particularly SKOV3-MR-1-shDNA-442) compared with SKOV3-shDNA-mock cells (Figure 5A-C). Both the rate of MR-1-shDNA-transfected SKOV3 cell proliferation and the degree of adhesion decreased, and the vigor of the cells was weakened (Figure 5D). Non-transfected SKOV3 cells and SKOV3-shDNA-mock cells began to spread 60 min after plating and became flattened by 120 min; however, the spreading of MR-1-shDNA-442 and parental cells was significantly delayed. These results indicate that MR-1 plays an important role in ovarian cancer cell adhesion, spreading and invasion.

### Role of MR-1 in Anti-Cancer Drug Therapy

**Figure 6:**
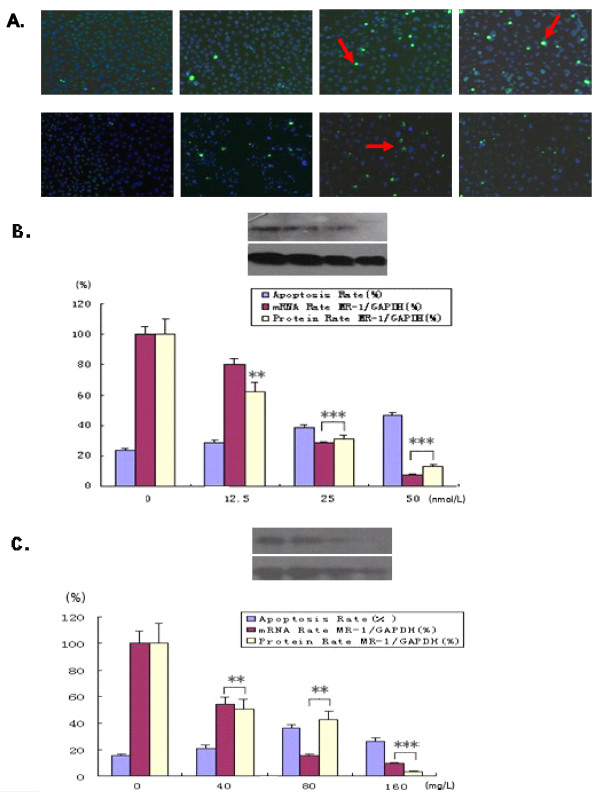
**Relationship between MR-1 expression levels and cell apoptosis**. (A) The degree of apoptosis in SKOV3 cells treated by the anti-tumor drugs, paclitaxel and carboplatin, is shown (apoptotic cells appear as bright green spots, some of which are highlighted with arrows). *Upper panel*: a-d show SKOV3 cells treated for 48 h with 0, 12.5, 25, and 50 nmol/L paclitaxel. *Lower panel*: e-h show SKOV3 cells treated for 48 h with 0, 40, 80 and 160 mg/L carboplatin. (B, C) The rate of apoptosis was associated with MR-1 gene and protein expression levels. Representative example from three independent experiments is shown. **P < 0.05; ***P < 0.01 compared with the untreated group (one-way ANOVA).

To identify whether MR-1 is a potential therapeutic target, SKOV3 cells were treated with different concentrations of the anti-cancer drugs, paclitaxel and carboplatin. Untreated SKOV3 cells grew as usual, with a spear-like shape. After treatment with paclitaxel or carboplatin for 48 hours, the number of cells decreased slightly and showed morphological changes. As the drug concentrations increased, the cells gradually shrank and their nuclei developed apoptosis-like changes, such as the appearance of green TUNEL-positive cores and the aberrant accumulation of granules within the nuclei (Figure 6A). MR-1 expression decreased in a dose-dependent manner at both the mRNA and protein levels following treatment with paclitaxel or carboplatin. Flow cytometry showed that the level of apoptosis also increased with increasing paclitaxel or carboplatin concentrations; although the degree of apoptosis decreased in cells treated with carboplatin at 160 mg/L, both mRNA and protein levels of MR-1 increased (Figure 6C).

## Discussion

Cancer progression and metastasis is a highly complex multi-stage process involving alterations in gene expression, increased cell adhesion, and changes in cell motility. Cancer cells move within tissues during invasion and metastasis, and cell invasion involves multiple processes that are regulated by various molecules [14]. Ovarian cancer has a high mortality, in large part owing to fact that the cells migrate and metastasize. This is a synergistic action associated with multiple factors, including overexpression of related genes, inhibition of tumor suppressor genes, and a failure to regulate cell proliferation. Ovarian cancer is a major health care issue worldwide; therefore, a sensitive biomarker that can detect ovarian cancer at the curative stage is urgently needed. Based on the finding that MR-1 plays a role in promoting the proliferation and invasion in some types of human cancers [6,12], and following our successful identification of the association between human epididymis protein 4 and ovarian cancer [15], we designed this study to comprehensively investigate the association between MR-1 and ovarian cancer and the roles it may play in pathogenesis or disease progression.

There are no reports examining the role of MR-1 in ovarian cancer. This is the first study showing that expression of MR-1 is increased in ovarian cancer tissues at both the mRNA and protein levels compared with benign control tissues. The immunohistochemistry results show that MR-1 is abundantly expressed in tumor tissues from ovarian cancer patients, particularly those with serous papillary ovarian cancer (Figure 3B), and that MR-1 is expressed in the cytoplasm of ovarian cancer cells (though it is normally localized to the nuclear membrane). To further examine the expression of MR-1 in ovarian cancer cells, RT-PCR and western blotting were used to analyze the ovarian cancer cell line, SKOV3. SKOV3 is derived from a human ovarian serous cystadenocarcinoma, which accounts for over 70% of ovarian cancers. The results showed that this cell line constitutively expresses high levels of MR-1 (Figure 1A and 2D).

MR-1-stably-transfected 239T cells were established to investigate the potential role of MR-1 in cell invasion and tumor progression (Figure 4B and 4C). These cells were used in Matrigel assays to examine cell adhesion and invasion. MR-1-stably-transfected cells showed increased aggregation and secreted proteinases that degrade constituents of the extracellular matrix. They also migrated faster than mock control cells. This suggests that MR-1 may play a role in cancer progression by regulating cell adhesion and motility. To confirm this, MR-1 expression by SKOV3 cells was knocked down by stable transfection of specific shDNAs. As expected, SKOV3 cells overexpressing MR-1 shDNA (known to significantly knock down the expression of MR-1) showed decreased adhesion, reduced vigor, and delayed spreading compared with cells transfected with mock-shDNA (Figure 5D). This further suggests that MR-1 plays an important role in ovarian cancer cell adhesion, spreading and invasion.

To assess whether MR-1 is a potential therapeutic target, SKOV3 cells were treated with the anti-cancer drugs paclitaxel and carboplatin, both of which induce apoptosis and are commonly used to treat ovarian cancer [16]. The number of apoptotic cells increased and MR-1 mRNA and protein levels decreased with increasing doses of either paclitaxel or carboplatin, strongly indicating that these anti-cancer drugs exert their therapeutic effects by antagonizing the effects of MR-1. Thus, MR-1 is potentially a novel therapeutic target for the treatment of ovarian cancer.

## Conclusion

This study shows that MR-1 is overexpressed in ovarian cancer tissue and cell lines. Knockdown of MR-1 expression inhibits cell adhesion and invasion, and anti-cancer drugs decrease the expression levels of MR-1 in cancer cells. Thus, MR-1 may be a novel biological marker and potential therapeutic target for the treatment of ovarian cancer. It could also be used to monitor the effect of anti-cancer therapies. Further studies are needed to clarify whether MR-1 is an early diagnostic marker for ovarian cancer and to develop its full therapeutic potential.

## List of abbreviations

MR-1: myofibrillogenesis regulator 1; PcAb: polyclonal antibody; RT-PCR: reverse-transcription polymerase chain reaction; ELISA: enzyme-linked immunosorbent assay; shDNA: short hairpin DNA; MLC2: myosin light chain-2.

## Competing interests

The authors declare that they have no competing interests.

## Authors' contributions

RQL designed the study, detected MR-1 expression levels in the transduced cells, measured cell adhesion and invasion, and drafted the manuscript. MS collected all the tissue samples and analyzed the expression levels of MR-1. JJF produced the MR-1 recombinant protein and anti-MR-1 antibodies and performed immunohistochemical staining. XG identified and characterized apoptosis induced by the anti-cancer drugs and edited the final manuscript. LG conceived of and supervised the project and reviewed the manuscript. All authors have read and approved the final manuscript.

## Pre-publication history

The pre-publication history for this paper can be accessed here:

http://www.biomedcentral.com/1471-2407/11/270/prepub
